# Exceptional Low-Temperature CO Oxidation over Noble-Metal-Free Iron-Doped Hollandites: An In-Depth Analysis of the Influence of the Defect Structure on Catalytic Performance

**DOI:** 10.1021/acscatal.1c04954

**Published:** 2021-12-01

**Authors:** Isabel Gómez-Recio, Huiyan Pan, Alberto Azor-Lafarga, María Luisa Ruiz-González, María Hernando, Marina Parras, María Teresa Fernández-Díaz, Juan J. Delgado, Xiaowei Chen, Daniel Goma Jiménez, David Portehault, Clément Sanchez, Mariona Cabero, Arturo Martínez-Arias, José M. González-Calbet, José J. Calvino

**Affiliations:** † Departamento de Química Inorgánica, Facultad de Químicas, Universidad Complutense, 28040 Madrid, Spain; ‡ Departamento de Ciencia de los Materiales e Ingeniería Metalúrgica y Química Inorgánica, Facultad de Ciencias, Universidad de Cádiz, Campus Rio San Pedro, Puerto Real 11510, Spain; § Institut Laue-Langevin, 38042 Grenoble cedex 9, France; ∥ Sorbonne Université, CNRS, Collège de France, Laboratoire Chimie de la Matière Condensée de Paris, 4 Place de Jussieu, 75005 Paris, France; ⊥ ICTS ELECMI-Centro Nacional de Microcopia Electrónica, Universidad Complutense, 28040 Madrid, Spain; # ICTS ELECMI-DME Universidad de Cádiz, Campus Rio San Pedro, Puerto Real 11510, Spain; ∇ Instituto de Catálisis y Petroleoquímica, CSIC, Marie Curie 2, Cantoblanco, 28049 Madrid, Spain

**Keywords:** hollandites, Fe modification, CO oxidation, defect structure, atomic scale analysis

## Abstract

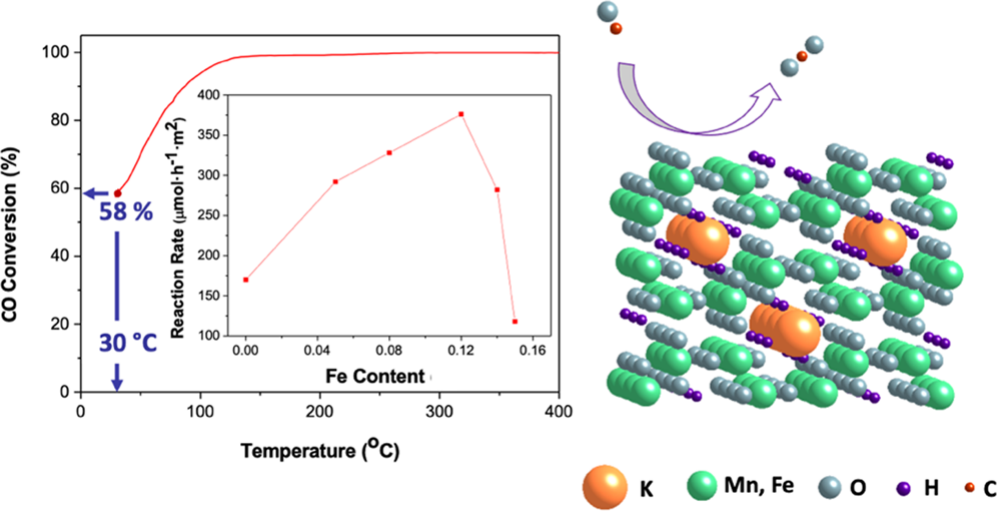

A family of iron-doped manganese-related hollandites, K*_x_*Mn_1–*y*_Fe*_y_*O_2−δ_ (0 ≤ *y* ≤ 0.15), with high performance in CO oxidation have been prepared. Among them, the most active catalyst, K_0.11_Mn_0.876_Fe_0.123_O_1.80_(OH)_0.09_, is able to oxidize more than 50% of CO at room temperature. Detailed compositional and structural characterization studies, using a wide battery of thermogravimetric, spectroscopic, and diffractometric techniques, both at macroscopic and microscopic levels, have provided essential information about this never-reported behavior, which relates to the oxidation state of manganese. Neutron diffraction studies evidence that the above compound stabilizes hydroxyl groups at the midpoints of the tunnel edges as in isostructural β-FeOOH. The presence of oxygen and hydroxyl species at the anion sublattice and Mn^3+^, confirmed by electron energy loss spectroscopy, appears to play a key role in the catalytic activity of this doped hollandite oxide. The analysis of these detailed structural features has allowed us to point out the key role of both OH groups and Mn^3+^ content in these materials, which are able to effectively transform CO without involving any critical, noble metal in the catalyst formulation.

## 1 Introduction

Manganese oxides are active catalysts in several redox processes with environmental interest, such as the oxidation of CO^1−4^ and volatile organic compounds (VOCs),^5−9^ or the selective reduction and storage reduction of NO*_x_* (SCR, NSR).^10−13^ Likewise, they have also demonstrated remarkable performances as electrode materials for the oxygen reduction reaction (ORR) for energy applications.^14−17^ Their nontoxicity, low cost, and large availability contribute also to their interest in such applications.

**Figure 1:**
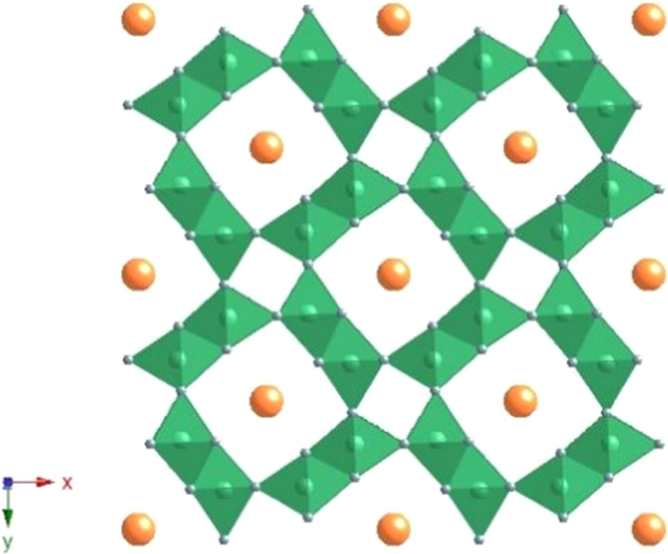
Schematic drawing of the crystal structure of hollandite oxide (K*_x_*MnO_2_). Color code: K, orange; Mn, green; and O, gray.

The A*_x_*MnO_2_-related hollandite stands as one of the most studied manganese oxides. The structure of this oxide^18^ is formed by dimers of edge-sharing MnO_6_ octahedra, corner-linked to other dimers in the *ab* plane, giving rise to 2 × 2 and 1 × 1 tunnels along the *c*-axis (Figure 1). Commonly, the smaller 1 × 1 tunnels are empty, while large A^z+^ cations, such as K^+^, Ba^2+^, Ag^+^, Pb^2+^, NH_4_^+^, or H_3_O^+^, are usually hosted in the bigger 2 × 2 tunnels together with some water molecules.^19−21^ The incorporation of these species in varying amounts within the channels leads to a mixture of manganese oxidation states (Mn^3+^ and Mn^4+^)^22^ to compensate the extra positive charge.

Driven by their outstanding performances in several catalytic processes, intense research has been devoted to tune the properties of hollandites using different strategies, such as modifying the nature of the A species, adding dopants, or changing the particle morphology and size.^11,16,17,23−28^ Recently, Pan et al. have evidenced in a systematic study of the K^+^ incorporation into the channels of K*_x_*MnO_2_ that large loadings of this alkaline cation lead to a lower reducibility using both H_2_ and CO, as well as lower activities in CO oxidation.^29^

Another approach—the most popular one—to tune the catalytic properties of hollandites consists in the partial substitution of manganese by other transition-metal cations,^30,31^ such as Cr^3+^, Fe^3+^, Co^2+^, Ni^2+^, Cu^2+^, or V^5+^. Especially, the incorporation of alio-cations modifies the concentration of anionic vacancies, which affects the structural oxygen mobility^11,23,30,32−38^ and, potentially, influences the catalytic activity of these materials.^11,24,39,40^ Another structural feature playing a role in the catalytic properties of doped hollandites is the location of the alio-cations, particularly whether they accumulate into the channels or, instead, as manganese substitute in octahedral positions. To date, this central question of the defect structure has been addressed only through indirect techniques that do not provide an unambiguous answer in the case of nanocrystalline doped hollandites.^23,41−43^

Iron holds a special role among dopants of hollandites. Its nontoxicity, abundance, and availability are similar to those of manganese. Its oxides hold manifold catalyst applications, such as oxidation of organic compounds, OER electrocatalysis, Fischer–Tropsch, and Fenton reactions, among others.^44−51^ In addition, iron is one of the rare metals giving rise to a hollandite-type structure, β-FeOOH,^52^ which anticipates the occurrence of quite specific structural effects when doping Mn hollandite with Fe. In spite of these considerations, the effect of iron as a dopant in K_x_MnO_2_ hollandite has only very scarcely been investigated,^53−58^ particularly attending to the ultimate effects of this modification in terms of the structural and electronic states of the solid.

Thus, Said et al.^42^ have reported an enhancement of the benzyl alcohol oxidation in gas phase after modification of a K hollandite with Fe in the molar content range from 0 up to 10%. However, essential chemical and structural characterization details missing in this contribution preclude a sound correlation between performance and structure. Specifically, the actual K and Fe contents in the doped samples were not reported. Likewise, the location of Fe within the structure was not properly supported by direct evidences. Finally, a quantification of the oxygen stoichiometry is also missing, which does not allow discussing the role of oxygen vacancies.

Positive effects on the catalytic performance induced by Fe modification of K*_x_*MnO_2_ have been also reported in other redox processes, like SO*_x_* removal,^57^ total benzene oxidation,^53^ or SCR of NO*_x_* by NH_3_.^54^ However, the level of structural characterization in these works is very limited and insufficient to analyze the contribution of the defect structure of the modified oxides.

Finally, in a study of the influence of Mn hollandite modification with different metals (Ce, Co, and Fe), Ma et al. reported a detrimental influence of both Co and Fe doping in the catalytic decomposition of ozone, which was attributed to a decrease in the Mn^3+^ content.^58^ In this contribution, all of the catalysts were modified to incorporate roughly a 12 mol % of Fe, Co, or Ce, which precluded an analysis of the influence of dopant loading.

The present work focuses on an in-depth, systematic, study of the ultimate structural aspects of iron-doped hollandites, with general formula K*_x_*Mn_1–*y*_Fe*_y_*O_2−δ_ (0 ≤ *y* ≤ 0.15), and their influence on the activity in a model catalytic reaction, CO oxidation. Particular attention is paid to identifying the conditions in which these materials provide their best performances. We then establish structure–property correlations that could be used to provide the basic guidelines for the design of new promising catalysts.^4,59^

## 2 Experimental Section

Hollandite-type manganese oxides were prepared by a synthetic pathway described in a previous work.^60^ This approach involves a one-pot synthesis in which the hollandite oxide is obtained by oxidation of Mn(SO_4_)_2_ with KMnO_4_, in acid medium. The iron-doped hollandites were prepared by adding a second metal precursor, Fe(NO_3_)_3_·9H_2_O, to the solution. The careful choice of the experimental conditions allowed us to obtain pure hollandites, avoiding the presence of other MnO_2_ polytypes, as well as structurally pure iron-doped hollandites, with general formula K*_x_*Mn_1–*y*_Fe*_y_*O_2−δ_ (0 ≤ *y* ≤ 0.15). To facilitate sample identification through the manuscript, we coded them using the molar Fe/Mn ratio in solution. For instance, an iron-doped hollandite prepared from a solution with a Fe/Mn molar ratio of 5:95 is coded as H5Fe, while the undoped material is referred as to H. In addition, an H15Fe sample, analyzed by neutron diffraction (ND), was prepared following a two-step route described in a previous work.^60^ Both the one-pot and the two-step products are structurally analogous (Table 1 and Figure S1). With the aim of simplifying the discussion, just the one-pot product data are discussed in this work, with the exception of those coming from the ND study.

**Table 1:** Chemical Composition as well as Cell Parameters Provided by Rietveld Refinement of Neutron Powder Diffraction, Le Bail Analysis of Powder X-ray Diffraction, and Cationic Oxidation State Composition Estimated by ND of Iron-Doped Hollandites (*Experimental Error of This Technique Is in the Order of 1%)^a^

		XRD				ND
		*a* (Å)	Χ^2^	composition	source (ref)	*a* (Å)
	chemical composition (EMPA)	*c* (Å)				*c* (Å)
**H**	K_0.12_MnO_δ_	9.8326(4)	2.33	K_0.11_Mn_0.81_^4+^Mn_0.19_^3+^O_1.96_	ND	9.8099(2)
		2.85080(9)			(60)	2.85354(5)
**H5Fe**	K_0.12_Mn_0.95_Fe_0.05_O_δ_	9.8533(3)	5.66	K_0.11_Mn_0.69_^4+^Mn_0.26_^3+^ Fe_0.05_^3+^O_1.90_	ND	9.8140(3)
		2.85509(6)			(60)	2.85555(6)
**H10Fe**	K_0.10_Mn_0.92_Fe_0.08_O_δ_	9.8673(4)	3.01	K_0.10_Mn_0.64_^4+^Mn_0.28_^3+^Fe_0.08_^3+^O_1.87_	this work^b^	
		2.85455(8)				
**H15Fe**	K_0.09_Mn_0.88_Fe_0.12_O_δ_*	9.8709(5)	2.31	K_0.11_Mn_0.58_^4+^Mn_0.30_^3+^Fe_0.12_^3+^O_1.80_(OH)_0.09_	ND	
		2.8566(1)			this work	
	K_0.12_Mn_0.91_Fe_0.09_O_δ_^**^	9.8614(4)	2.24			9.8381(4)
		2.85881(9)				2.86373(9)
**H20Fe**	K_0.07_Mn_0.86_Fe_0.14_O_δ_	9.8365(8)	2.19	K_0.09_Mn_0.72_^4+^Mn_0.13_^3+^Fe_0.15_^3+^O_1.904_	ND	9.8659(7)
		2.8543(2)			(60)	2.86568(1)
**H25Fe**	K_0.04_Mn_0.85_Fe_0.15_O_δ_	9.829(1)	1.25	K_0.04_Mn_0.72_^4+^Mn_0.13_^3+^Fe_0.15_^3+^O_1.88_	this work^b^	
		2.8598(6)				

a ND: composition determined from neutron diffraction.

b The cationic composition corresponds to that determined by EMPA, and the Mn^3+^ content was interpolated in these cases using the Mn^3+^ vs Fe^3+^ dependency observed in the samples analyzed by neutron diffraction (see Figure S3). Chemical composition of one-pot* and two-step^**^ H15Fe samples.

X-ray diffraction (XRD) patterns were recorded in a PANalytical X’Pert PRO MPD diffractometer, equipped with an X-ray source worked with Cu Kα radiation at 45 kV and 40 mA. Patterns were recorded in the 2θ range of 5–120°, with a step size of 0.017° and a collection time of 100 s with an X’Celerator fast detector.

Neutron diffraction analysis was performed on a D2B diffractometer (λ=1.594 Å),^61^ at Institut Laue Langevin, Grenoble (France). The patterns were recorded in the 2θ range of 0–160°, with a step size of 0.05°. The data were then analyzed by the Rietveld^62^ method using the FullProf^63^ software. Before data collection, the samples were dried for 12 h at 120 °C, with the aim of avoiding a high background associated with the incoherent scattering of hydrogen.^64^ According to XRD (Figure S2), samples after the drying process remain structurally unchanged, in comparison with humid counterparts.

Cation compositional analysis was performed by electron microprobe analysis (EMPA) with a JEOL Superprobe JXA-8900M instrument, equipped with five wavelength-dispersive X-ray spectrometers, analyzing a total of 20 areas 5–10 μm large.

The study of the manganese and iron oxidation states was performed in a probe spherical aberration-corrected JEOL JSM-ARM200F (Cold Emission Gun) microscope working at 80 kV using a probe size of ∼0.08 nm and a low current emission density to minimize damage of the samples under the electron beam. Inner and outer collection semiangle values of 68 and 280 mrad, respectively, were set for the acquisition of atomically resolved HAADF images. The microscope is equipped with a GIF-Quantum ERTM spectrometer, which was used for electron energy loss spectroscopy (EELS) with a collection semiangle of 18 mrad and a convergence semiangle of 20.3 mrad. The samples were deposited directly onto holey-carbon Cu grids to avoid contact with any solvent. The L_2,3_ ratio values were calculated from both the second derivative of the EEL spectra and using a Hartree–Slater step function to remove the continuum contribution. Both methods lead to comparable results, but according to the procedure described by Varela et al.,^65^ we present the results obtained using the latter approach. The EEL spectra were acquired using the spectrum line mode, with an energy dispersion of 0.25 eV per channel, an acquisition time of 0.5 s over an average total number of 40–100 points (depending on the number of layers), and a pixel size of 1.5 Å. Principal component analysis (PCA) was performed on the EELS datasets to de-noise the spectra, using the MSA plug-ins for Gatan DMS analysis toolbox.^66^ A total of 21 spectrum lines were acquired, from which the average oxidation was estimated.

The BET surface area was determined by N_2_ physisorption isotherms at 196 °C using an Autosorb iQ3 equipment. Prior to analysis, the samples were outgassed at 300 °C for 8 h.

CO oxidation tests were performed in a tubular U-shaped quartz reactor, which was loaded with a mixture of 25 mg of the samples and 50 mg of silicon carbide, to avoid hot spots in the catalytic bed during the reaction. The reactor was heated up to 600 °C (heating rate 10 °C·min^–1^) under 100 mL·min^–1^ of feed gas composition 1% CO, 0.6% O_2_, and 98.4% He. The flow was adjusted with Bronkhorst mass-flow controllers, and the outlet gasses were analyzed with a Pfeiffer Vacuum Thermostar GSD301T1 mass spectrometer. Before the CO oxidation test, all of the samples were pretreated for 1 h in a 60 mL·min^–1^ gas flow of 5% O_2_/He. To study the influence of the activation conditions on catalytic activities of the materials, different activation temperatures were used, which were chosen on the basis of previous temperature-programmed oxidation (TPO) experiments in the same gas flow, 60 mL·min^–1^, 5% O_2_/He.

Temperature-programmed reduction and oxidation (TPR and TPO) were performed in an experimental setup analogous to that used in the CO oxidation test. In all of these experiments, 75 mg of sample was heated from room temperature up to 900 °C, using a heating ramp of 10 °C·min^–1^. TPO experiments were performed under a flowing mixture of 5% O_2_/He without any prior pretreatment of the samples. CO-TPR and H_2_-TPR were carried out in a similar way but under 5% CO/He and 5% H_2_/Ar flows, respectively. Moreover, before the TPR tests, all of the samples were pretreated 1 h at 350 °C in a 60 mL·min^–1^ gas flow of 5% O_2_/He.

For quantitative purposes, additional H_2_-TPR tests were performed in an AutoChem II 2920 automated characterization system, equipped with a calibrated thermal conductivity detector (TCD). In these essays, 50 mg of sample was loaded into a quartz reactor and heated from room temperature up to 900 °C at a heating rate of 10 °C·min^–1^. The mixture gas used had the same flow and composition described above. In all TPR tests, the samples were pretreated for 1 h at 350 °C in 60 mL·min^–1^ of feed gas composition 5% O_2_ and 95% He.

In all of the experiments using mass spectrometry as the analysis technique, the different mass/charge (*m*/*z*) signals were normalized with respect to that corresponding to the inert gas present in the gas mixture with the goal of improving the S/N ratio and avoiding artifacts related to pressure changes inside the mass spectrometer chamber.

Pearson correlation analysis as well as related studies were performed considering the iron content determined by EMPA.

The DRIFTS spectra were carried out using a Harrick cell (in which ca. 100 mg of sample was introduced) and a Bruker Equinox 55 FTIR spectrometer with an MCT detector. The spectra were collected with 20 scans in reflectance units and were further transformed to Kubelka–Munk units. The sample was subjected to pretreatment under 5% O_2_/He for 1 h at 350 °C and then, after cooling to room temperature, to a reactant stream of CO+O_2_ (1% CO + 0.6% O_2_ in He, with a total flow of 100 mL min^–1^) from room temperature to 300 °C (in steps, to achieve steady conditions on the basis of the absence of changes in the spectra recorded at every reaction temperature), similar to experiments done for catalytic activity tests. Activity data were collected by mass spectrometry (MS, Pfeiffer Omnistar) coupled in line with the DRIFTS cell. Two runs were done for each of the two examined samples, which demonstrated the reproducibility of the results obtained.

## 3 Results and Discussion

The stabilization of hollandite-type single phases was confirmed by both XRD and ND data. The cell parameters of all samples were calculated by the Le Bail analysis of the XRD data (S.G. *I*4/*m*, Figure S2 and Table 1). As the dopant concentration increases, the diffraction peaks become broader, suggesting a decrease of the particle size as a consequence of the dopant incorporation, in good agreement with previous works.^58,60^

The metal content of all of the catalysts was determined by electron microprobe analysis (EMPA). Table 1 indicates the presence of iron and Mn. The K/(Fe + Mn) molar ratio remains nearly constant, around 0.10, up to 15 mol % Fe. The Mn content decreases with the increase of the Fe content. The actual Fe/Mn ratio in the sample is lower than the corresponding reactants ratio. This loss of Fe is attributed to the partial dissolution of Fe during synthesis and is supported by the color of the supernatant liquid after the separation of the solid. Both EMPA and cell parameter evolution with doping suggest that Fe replaces Mn in the oxide lattice.^67^ According to the literature, Fe^3+^ would substitute Mn^3+^ in hollandite-type structure;^58^ however, our results suggest that Fe^3+^ could also substitute Mn^4+^ since, as shown below, oxygen deficiency is also observed.^60^

We analyzed most of the samples by neutron diffraction to get a deeper insight into their structure and composition, particularly Mn/Fe ratios, oxygen content, and, therefore, average Mn oxidation state (Table 1). The details of the analysis are described elsewhere,^60^ except for the **H15Fe** sample, which we present herein. Note, first, the good agreement between EMPA and ND results, which differ by only ±1%.

**Figure 2:**
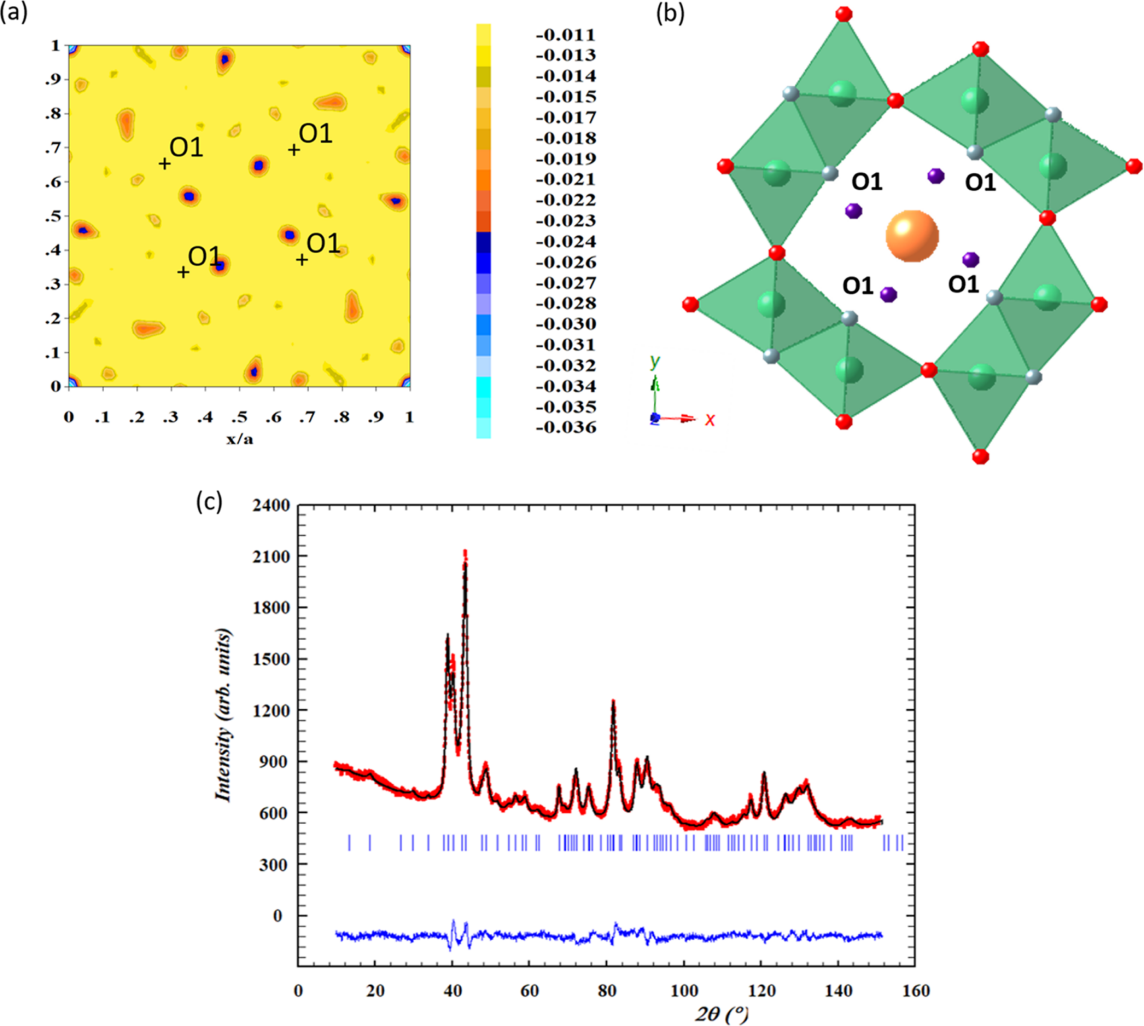
Structural analysis by powder neutron diffraction of the iron-doped **H15Fe** sample. (a) Difference Fourier map for neutron scattering density of the section *z* = 0.5. The map shows the positions in which the most prominent negative scattering density appears. (b) Schematic drawing of the crystal structure and (c) Rietveld refinement of neutron powder diffraction data for K_0.11_Mn_0.877(2)_Fe_0.123(2)_O_1.80(1)_(OH)_0.09(1)_ The observed patterns (red circles), calculated patterns (continuous black line), and the difference curves (continuous blue line) are shown. Color code: K, orange; Mn, green; O1, gray; O2, red; and H, purple.

Importantly, while the Rietveld refinement of the ND data shows that the Fe-modified materials are isostructural with the undoped one, the difference Fourier maps clearly depict a negative scattering density close to the O1 oxygen position (*x* ∼ 0.44, *y* ∼ 0.35, *z* = 0.5), as shown for the **H15Fe** sample (Figure 2a). This residual negative density can account for hydrogen atoms bonded to O1 (Figure 2b). As a consequence, a new refinement was carried out by considering the H atoms at the 8h site to complete the structural model. All atom positions, overall temperature factor, Mn/Fe, and oxygen occupancies were refined (see the SI). The potassium content was fixed according to the value obtained by EMPA because its large mobility inside the tunnels gives rise to a large isotropic thermal parameter that yields instabilities during the refinement. Figure 2c shows the results of the final ND data fitting, whereas the corresponding structural parameters and distances are included in Tables S1 and S2, respectively. The refinement of the oxygen occupancies shows the presence of anionic vacancies in O2 sites. The O1–H distance obtained, 1.09 Å, is in good agreement with those found in the isostructural β-FeOOH phase, 0.94 and 1.01 Å^68^ for the OH group located at the midpoints of the tunnel edges. This fact allows considering the presence of hydroxyl groups formed by the H–O1 bond in the **H15Fe** sample. Therefore, the hollandite structure is formed by M–(O,OH) octahedra sharing corners and the final composition obtained from ND is K_0.11_Mn_0.877(2)_Fe_0.123(2)_O_1.80(1)_(OH)_0.09(1)_. The M–O octahedra are distorted, with the average M–(O, OH) bond length (1.916 Å) very similar to that found in other compounds.^69,70^

It is worth noting the difference observed between the cell parameters obtained from XRD and ND datasets (Table 1). This difference is very likely related to the particular drying thermal treatment performed before the acquisition of ND data, which was necessary to avoid an intense background on the ND patterns due to the incoherent scattering of the protons of water molecules.^60,64^ In the pristine samples, without drying, probed by XRD, for instance, the **H15Fe** sample, water promotes an increase of the *a* cell parameter, probably because van der Waals interactions are enhanced by the hydroxyl groups.

According to the ND data (Table 1), the *a* cell parameter continuously increases with Fe content, in good agreement with the larger atomic radius of Fe(III), compared to Mn(IV), 0.65 and 0.53 Å, respectively.^67^ This increase is also consistent with the cell parameters of the end members of the homologous series: α-MnO_2_^71^ and β-FeOOH.^68^ As the *a* cell parameter reaches its greatest value for the hydroxylated compound **H15Fe** according to XRD, the presence of hydroxyl groups seems to stabilize a larger amount of water, probably because of hydrogen bonding interactions. Note that the **H15Fe** sample is the only one in which hydroxyl groups into the hollandite tunnels have been detected by ND. Interestingly, this observation could be related to the isostructural β-FeOOH, where hydroxyl groups are indeed located into the tunnels. In the remaining Fe-doped catalysts, these species could be present but in a much smaller amount or randomly distributed, below the detection limit of ND.

As already reported,^60^ the particle size is decreasing as the content of dopant increases (Figure S4).

Mn^3+^/Mn^4+^ ratios were calculated taking into account the electroneutrality for the compositions determined from ND. In any case, STEM-HAADF-EELS was also used to probe structural and compositional homogeneities (Figure S5) as well the oxidation states of the 3d metals.

**Figure 3:**
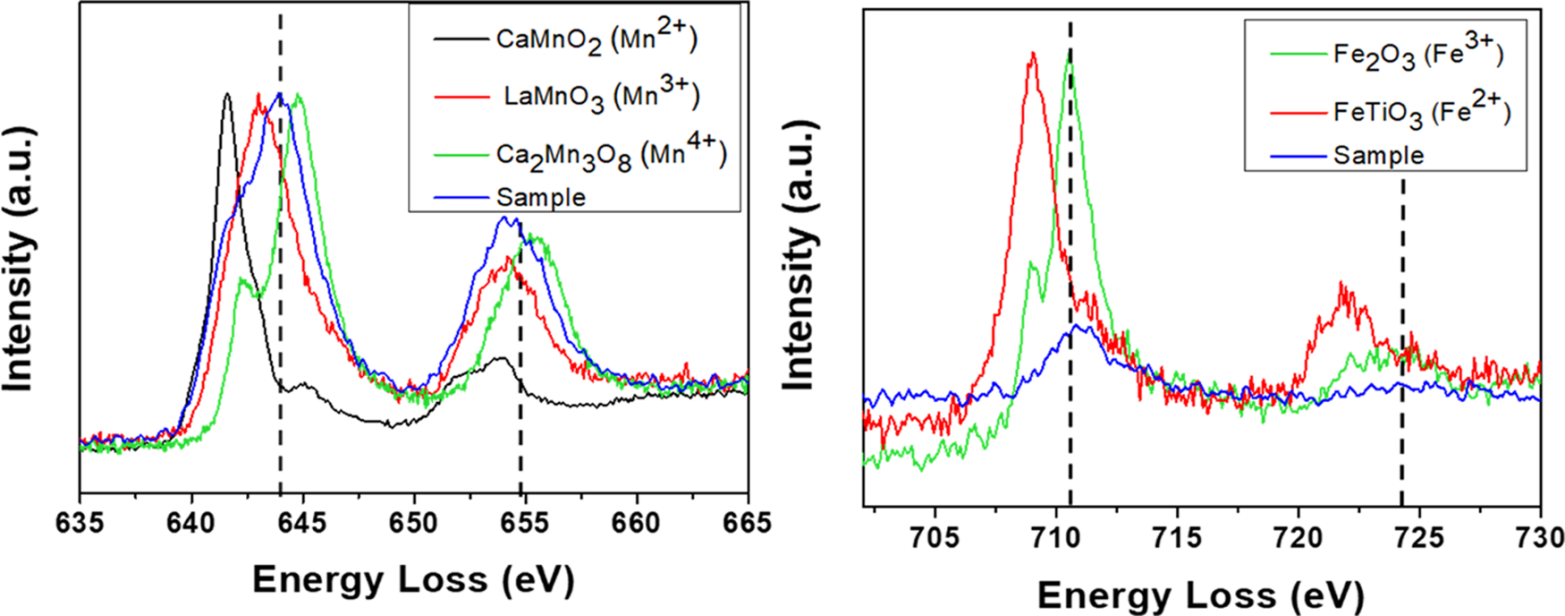
(a) Characteristic Mn L_2,3_ edge and (b) Fe L_2,3_ edges of sample **H15Fe** in comparison with standards.

Figure 3 shows the energy edge onsets for Mn-L_2,3_ and Fe-L_2,3_ for the **H15Fe** sample, in comparison with well-known standards. The Mn-L_2,3_ edge is in between the Mn^3+^ and Mn^4+^ standards, while the Fe-L_2,3_ edge fits in with the corresponding Fe^3+^ standard. Hence, Mn^3+^, Mn^4+^, and Fe^3+^ coexist in the same materials as we have already shown for the other doped samples.^60^

**Figure 4:**
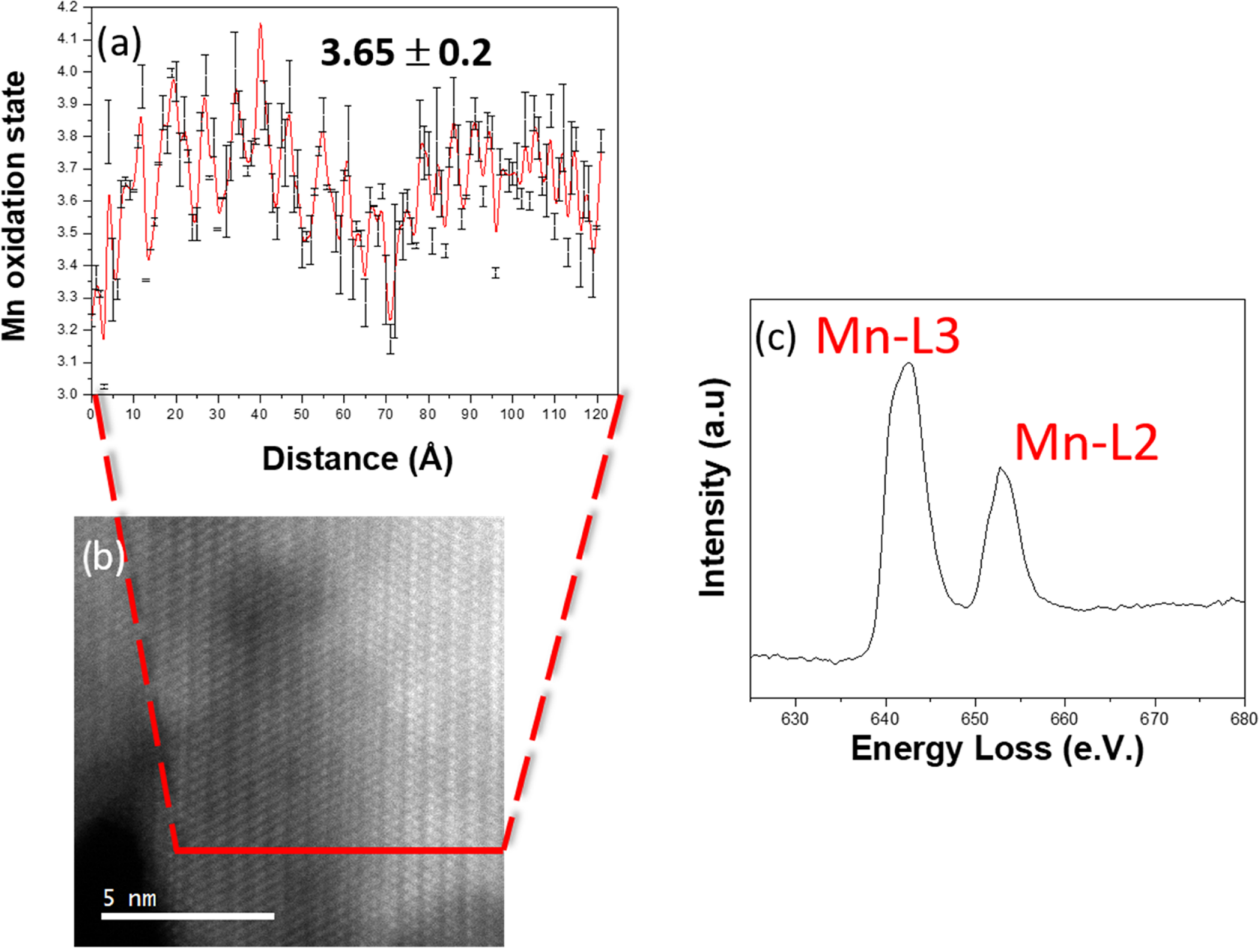
(a) Plot of the Mn oxidation state vs distance (error bars are included) obtained from an EEL line scan obtained on the H15Fe sample. Error bars were obtained by repeating the L_2,3_ ratio calculation but varying the position of the integration window of the original calculation and measuring the difference. (b) STEM-HAADF image shows the line along which the spectra were acquired, as well as (c) the corresponding sum spectrum of the Mn-L_2,3_ edge.

To get more precise information concerning the Mn oxidation state, its numerical value was estimated from the intensity ratio L2/L3 of the L2 and L3 lines (L_2,3_ ratio) in the EEL spectra, according to the procedure described by Varela et al.,^65^ who reported a linear relationship between the L_2,3_ ratio and the Mn oxidation state. The average oxidation state, gathered from the different line scans, oscillates in between 3.3 and 3.9. The average value of the above measurements is 3.65 ± 0.24. Figure 4 shows a representative graphic from one of the line scans performed during the experiment. Despite the high dispersion obtained from the EELS data, the numerical estimate of the Mn oxidation state sustains the previously reported coexistence of Mn^3+^ and Mn^4+^ in the sample. This result is also in good agreement with the average Mn oxidation state of +3.66 expected from the composition gathered by ND.

We have further investigated the location of Fe. The element distribution maps established from the EELS spectrum imaging experiments^59^ reveal that the dopant predominantly substitutes Mn in the octahedral positions, in good agreement with the results of the ND Rietveld refinements.

To complete the compositional characterization, the oxygen content was evaluated by neutron diffraction (Table 1).^72−75^ By combining these values with the Fe^3+^, Mn^4+^, and Mn^3+^ contents determined above, we could access the whole chemical composition of the iron-doped hollandite materials. The quantitative analysis of EELS experiments performed in spectrum line mode (Figure S6) evidences that composition remains roughly homogeneous throughout the crystallites, with variations from bulk to surface lying within the limits of the experimental error of this technique (∼5%).

**Figure 5:**
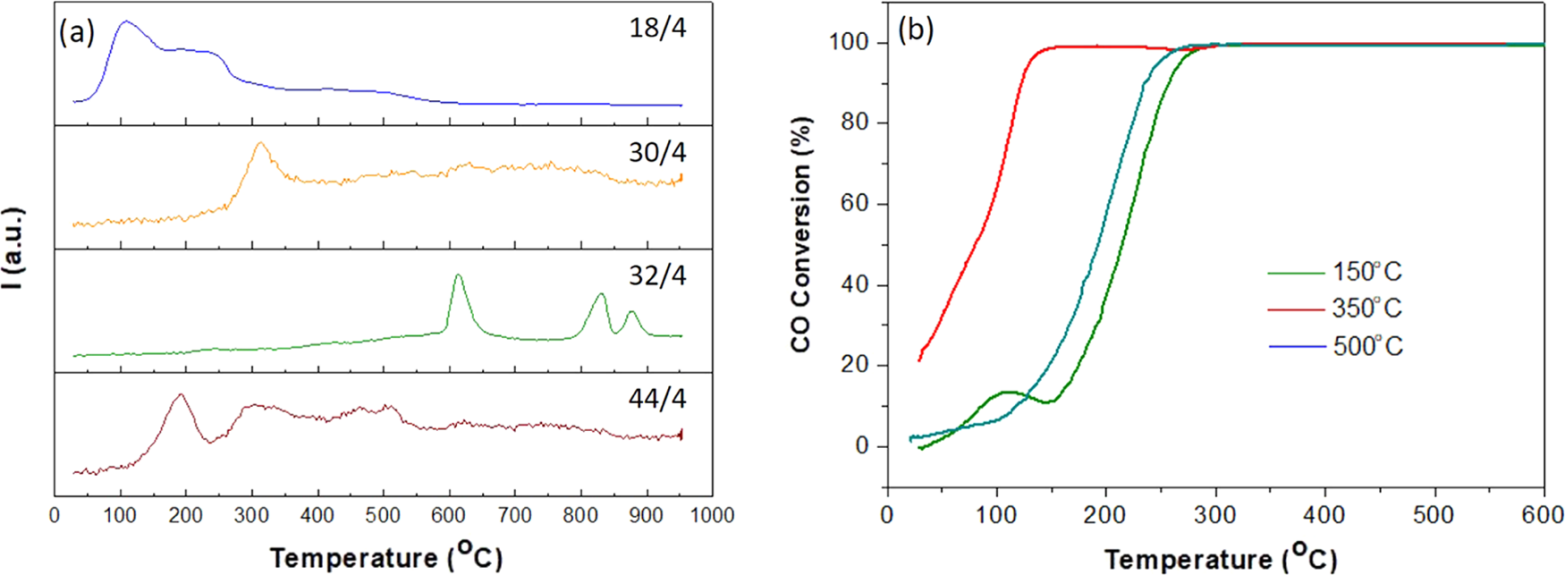
(a) TPO profiles of undoped Mn hollandite *m*/*z* = 18, 30, 32, and 44 account for water, NO, O_2_, and CO_2_ fragments, respectively. The MS signals were normalized to that of the carrier gas (helium). (b) Influence of pretreatment conditions on the CO oxidation behavior of undoped hollandite.

The catalytic properties of the doped hollandites have then been investigated for the CO oxidation reaction. The influence of catalyst pretreatment conditions on the chemical composition of the hollandites was first assessed by TPO. Figure 5a shows the most relevant mass/charge (*m*/*z*) ratios during a TPO of the undoped Mn hollandite. Note that the oxide dehydrates (*m*/*z* = 18) and decarbonates (*m*/*z* = 44) at low temperatures (*T* < 250 °C); the decomposition of nitrates originating from the initial iron salts (*m*/*z* = 30) takes place at medium temperatures (250 < *T* < 450 °C). Dehydroxylation takes place mostly in the 200–300 °C temperature range but extends in a rather wide tail up to roughly 500 °C. Finally, oxide reduction (*m*/*z* = 32) occurs only at high temperatures (*T* > 550 °C). The TPOs of the iron-doped hollandites present similar profiles (Figure S7). According to these analyses, all compounds are initially hydroxylated, at least on the surface.^32^ Moreover, the high-temperature tails observed in the *m*/*z* = 18 signal suggest the presence of a small fraction of more tightly bonded OH groups, which could be related to those detected in the channels of the H15Fe catalyst by ND.

We then evaluated the influence of the calcination pretreatment on the CO oxidation catalytic activity of the undoped hollandite (Figure 5b and Table 2). Calcination temperatures of 150, 350, and 500 °C were chosen to remove water, carbonates, and nitrates and hydroxyl groups, respectively. The best activity is achieved for the sample pretreated at 350 °C. Hence, nitrate groups block the active centers, but tightly bonded hydroxyl groups seem to play a role in promoting the catalytic activity. This positive impact of hydroxyl groups on the CO oxidation catalysis has been reported for a variety of oxides, enabling the formation of intermediates for CO oxidation at a low temperature.^76−79^

**Table 2:** Influence of the Pretreatment Temperature of the Undoped Hollandite Oxide on the CO Oxidation Temperatures for Different Conversions (*T*_*n*%_)

*T*_Pre-treatment_ (°C)	150	350	500
*T*_10_ (°C)	84		127
*T*_50_ (°C)	213	79	196
*T*_90_ (°C)	257	120	239

**Figure 6:**
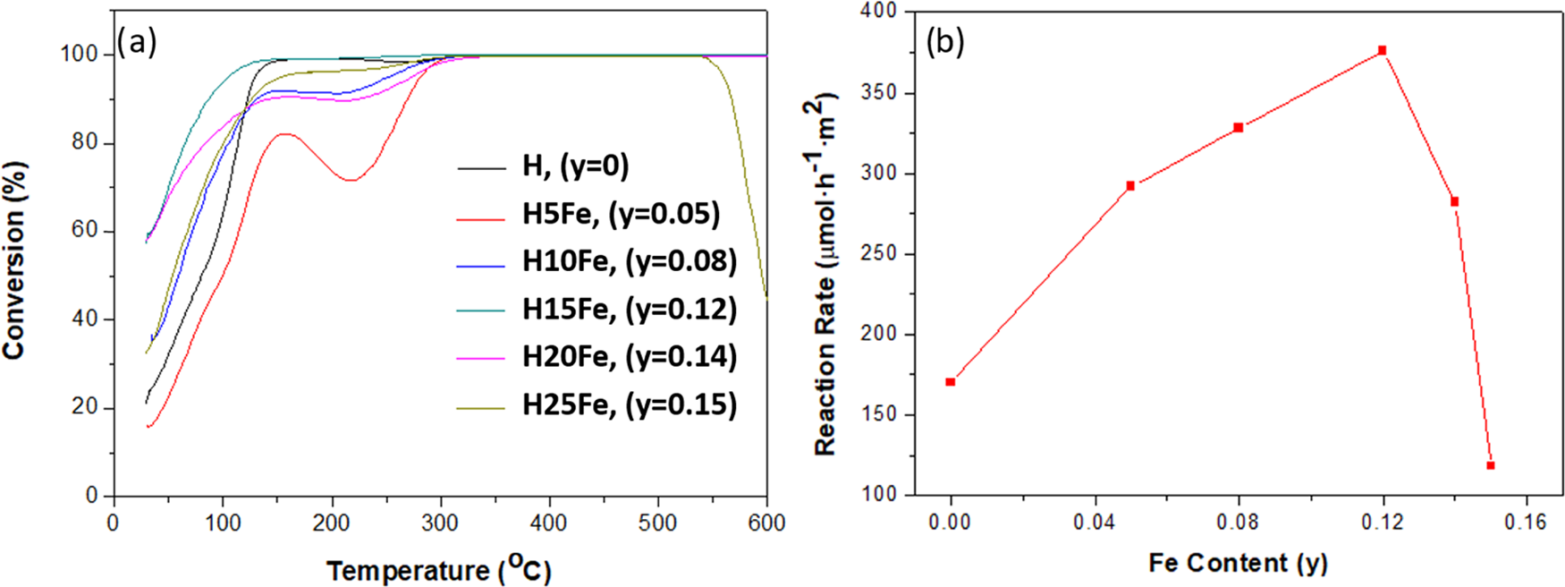
(a) CO oxidation performance and (b) reaction rate at room temperature as a function of the iron content *y* in K*_x_*Mn_1–*y*_Fe*_y_*O_2−δ_ oxides.

The same procedure was applied to the iron-doped catalysts (Figure 6a and Table 3). All doped samples oxidize more than 15% of CO at room temperature. Furthermore, **H15Fe** and **H20Fe** are exceptionally active since both are able to oxidize more than 55% of CO at room temperature (Conv,_RT_Table 3). To the best of our knowledge, no Fe-doped hollandites with such high activities have ever been reported. These materials depict light-off temperature, *T*_50_, and *T*_90_ values lower than those reported in previous works.^33,36,37,80^ However, the increase in the surface area observed for the compositions with higher dopant concentrations (Table 3) complicates the comparison of the activity of the different materials.

**Table 3:** CO Oxidation Parameters, *S*_BET_, and H_2_-TPR Results for the Whole Set of Hollandite Oxides (**H10Fe** and **H25Fe** Anionic Composition Is Denoted by δ since It Has Not Been Determined by ND)

	chemical composition	*T*_10_ (°C)	*T*_50_ (°C)	*T*_90_ (°C)	conv,_RT_ (%)	*S*_BET_ (m^2^·g^–1^)	rate (μmol CO·h^–1^ ·m^–2^)	*T*_α_ (°C)	H_2_ consumption (mmol·g^–1^)
H	K_0.11_MnO_1.96_		79	120	21	132	170	232	10.9406
H5Fe	K_0.11_Mn_0.95_Fe_0.05_O_1.90_		102	278	15	55	292	197	9.5847
H10Fe	K_0.10_Mn_0.92_Fe_0.08_O_1.87_		58	129	37	121	328	170	8.5849
H15Fe	K_0.11_Mn_0.88_Fe_0.12_O_1.80_(OH)_0.09_			85	57	162	376	108	8.0449
H20Fe	K_0.09_Mn_0.85_Fe_0.15_O_1.90_			138	58	220	282	174	8.4456
H25Fe	K_0.04_Mn_0.85_Fe_0.15_O_1.88_		52	124	32	291	118	118	9.3340

The activity curves of the doped samples show, particularly in the case of the H5Fe catalyst, a decrease in the CO conversion in the 150–200 °C temperature range, which is followed by a monotonous increase at higher temperatures until reaching 100% conversion. This suggests the occurrence of deactivation phenomena in this temperature range. Blockage of active sites by reaction spectators or, alternatively, modification of the redox state under working conditions could be at the roots of this behavior. A precise clarification of this particular aspect deserves an in-depth analysis, which is beyond the reach of the present contribution. Note in any case that the effect is particularly pronounced in H5Fe, the catalyst depicting the lowest specific surface area.

Focusing on the room-temperature performance and to compare the intrinsic activity of each composition, the reaction rate was evaluated (Table 3) considering the percentage of CO oxidized to CO_2_ at room temperature per surface area unit. Figure 6b indicates that the reaction rate follows a volcano shape when plotted against the Fe content and that the best performance is achieved for the **H15Fe** sample K_0.11_Mn_0.88_Fe_0.12_O_1.80_(OH)_0.09_. Therefore, Fe^3+^ does not seem to contribute directly as the catalytic site, as an increase in the Fe content beyond **H15Fe** does not correlate with an increase in the activity (Figure 6b).

In any case, the promoting effect of iron doping has been explored by DRIFTS in the most active sample H15Fe, as shown in the Supporting Information (Figure S8). The results show that this catalyst presents the capability to form carbonate species under reaction conditions that could open a new reaction path with an enhanced CO oxidation rate.

**Figure 7:**
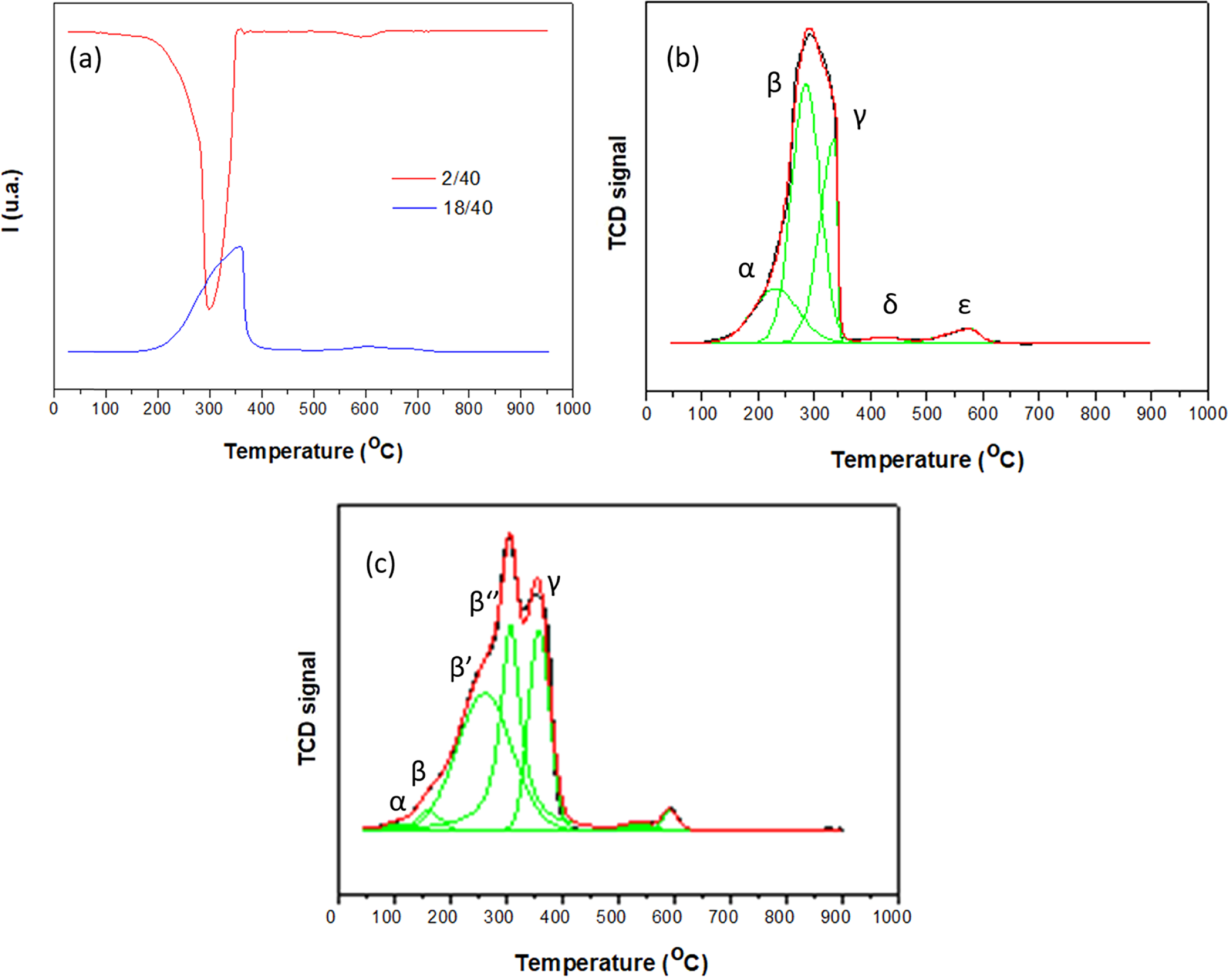
H_2_-TPR profiles of the undoped hollandite oxide with (a) mass spectrometry, (b) TCD detection, and (c) H_2_-TPR-TCD of **H15Fe**. The MS signals were normalized to that of the carrier gas (argon).

To better understand the oxygen exchange properties of these materials, the redox properties were characterized through TPR analysis under different reducing atmospheres, H_2_ and CO. Figure 7a,b shows H_2_-TPR profiles of the undoped hollandite followed by both mass spectrometry (MS) and thermal conductivity detector (TCD), respectively.

The most relevant *m*/*z* ratios were monitored by H_2_-TPR-MS first for the undoped hollandite (Figure 7a, *m*/*z* = 18 and *m*/*z* = 2). The *m*/*z* = 2 trace shows a broad peak of dihydrogen consumption centered at 300 °C. The good agreement with the water evolution signal (*m*/*z* = 18) suggests that the oxygen from the oxide is removed as water. The CO-TPR-MS study (Figure S11a) shows the evolution of CO_2_ in close agreement with the CO consumption. The complex shape of the CO peak suggests the overlap of different reduction events within the 150–400 °C temperature range. Likewise, a second reduction event, of a much lower intensity, is observed in the H_2_-TPR profiles at higher temperatures (>500 °C).

According to H_2_-TPR-TCD (Figure 7b and Table 3), the H_2_ consumption during reduction of the undoped hollandite was 10.94 mmol·g^–1^, very close to the theoretical value expected for the reduction of K_0.11_MnO_2_ (=K_0.11_Mn_0.89_^4+^Mn_0.11_^3+^O_2_, 10.96 mmol·g^–1^). Apart from intrinsic error sources, the accuracy in the quantification of the oxygen content values from the hydrogen consumption measured by H_2_-TPR-TCD values is limited by the hydration of the samples after preparation, as detected in the TPO experiments. Effectively, the incorporation of undetermined amounts of water within the channel structure of the hollandite may take place during the synthesis or after the exposure of the fresh sample to air. This water is eliminated during the treatment applied prior to the TPR but leaves some uncertainty concerning the total content of Mn in the sample. This could partly explain the slight difference between the oxygen contents determined from ND and the quantification of the H_2_-TPR-TCD. Despite this limitation, note that the evolution observed in the H_2_ consumption parallels that of the Mn^4+^ values obtained from ND.

Five reduction peaks (Figure 7b) can be distinguished upon heating, which have been labeled consecutively as α, β, γ, δ, and ε. The α peak at a low temperature (232 °C) can be related to the reduction of surface oxygen species, preserving the hollandite structure.^11,30,34,81^ The β and γ peaks at *ca.* 285 and 319 °C can be attributed to successive reductions from MnO_2_ → Mn_3_O_4_ → MnO, respectively, in agreement with their reported characteristic temperatures and with the measured area ratios β/γ = 2:1, a value in good agreement with the expected hydrogen consumptions at each step (Table S3).^11,35,82^ The β peak can be deconvoluted into two overlapping peaks (β and β′) (Table S3), respectively, very likely accounting for the presence of MnO_6_ octahedra with nonequivalent environments, due to differences in local concentrations of potassium or oxygen vacancies.^11^ On the other hand, the origins of δ and ε peaks are not well established to date. Jia et al. described a reduction process in γ-MnO_2_ at the same temperature as the δ event,^83^ while the ε peak, at the highest-temperature end, could be related to the reduction of potassium oxides. CO-TPR of undoped hollandite (Figure S11a) depicts only two broad asymmetric peaks at 230 and 396 °C, related with the β and γ Mn reduction events described above.^36,37^

The comparison between the H_2_-TPR profile of the undoped sample (Figure 7b) and of the doped oxides (Figure 7c and Figure S12) reveals that the α process is shifted toward lower temperatures in the doped materials (Table 3). This effect could be related to a higher mobility of surface oxygen as a consequence of either the observed increase of the oxygen vacancy content or the presence of Fe in the neighborhoods of Mn, as it has been reported for other oxides whose reducibility is improved when the vacancy content increases.^84^

On the other hand, for the **H5Fe, H10Fe**, and **H15Fe** samples, the β and γ reduction events are also shifted to lower temperatures (Figure S12), which suggests that the Mn–O bond weakens as a result of iron incorporation.^30^ Likewise, the intensity of the peak at a higher temperature increases due to the contribution of iron reduction.^85−87^

CO-TPR-MS results (Figure S11b) confirm the shift toward lower temperatures of the reduction process in doped materials, as well as the appearance of a new reduction peak at high temperatures.

**Figure 8:**
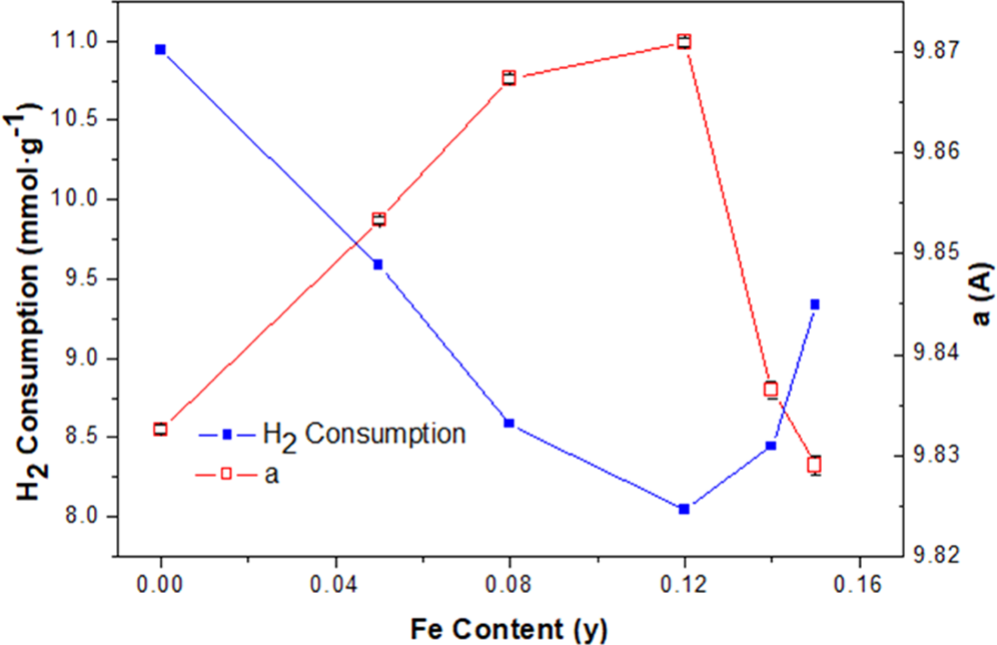
H_2_ consumption and *a* cell parameter against dopant composition (K*_x_*Mn_1–*y*_Fe*_y_*O_2−δ_). Error bars display error calculated by the Le Bail analysis.

These results suggest that iron incorporation promotes a decrease in the reduction temperature up to the **H15Fe** sample, which appears as the optimal composition. Higher iron concentrations lead to a higher temperature for the α reduction event (Table 3). It is important to highlight, however, that the total H_2_ consumption values in the H_2_-TPR-TCD experiments (Table 3) decrease continuously to a minimum for the K_0.11_Mn_0.88_Fe_0.12_O_1.80_(OH)_0.09_ (**H15Fe**) composition (Figure 8). Overall, the evolution of the oxygen exchange performance in these materials, both in terms of characteristic temperatures at which the reduction events take place and the total amounts of reductant involved in these events, can be related to two factors linked to the dopant incorporation: a lower average oxidation state of the cations and a higher amount of oxygen vacancies.

An in-depth interpretation of the relationship between catalytic performance and Fe doping becomes a challenging exercise given the intrinsic structural and compositional complexities as well as the variety of factors that may contribute to the final outcome.

**Figure 9:**
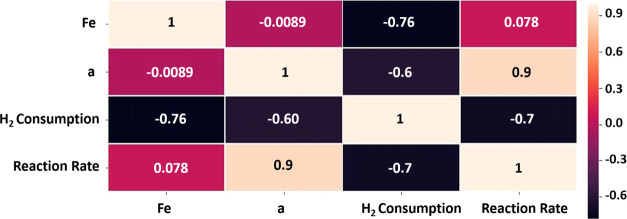
Pearson correlation matrix showing the relationship between different variable parameters.

Nevertheless, we can start with a Pearson cross-correlation analysis between the Fe content, CO oxidation behavior, hydrogen consumption, and XRD *a* cell parameter (Figure 9), which reveals the relationship between these parameters. In particular, the *a* cell parameter is highly correlated with reaction rate at room temperature (c.c. = 0.9), whereas total hydrogen consumption in TPR is strongly negatively correlated with the reaction rate at room temperature (c.c. = −0.7). Therefore, these results suggest that the factors responsible for the tunnel expansion (hydroxyl groups and Mn^3+^) can be related with the best performance achieved by these materials, while the accountable phenomena of a lower hydrogen consumption (lower average oxidation state of cations) contribute also to the activity of these oxides. It is important to highlight that these two effects, the presence of hydroxyl groups and oxidation state, are intimately related, as revealed by ND and EELS.

Variation of catalytic performance cannot be in this case directly related to the K content, since for the set of samples comprising H, H5Fe, H10Fe, and H15Fe, this parameter remains fairly constant, whereas activity grows continuously with the Fe content, till reaching a maximum at H15Fe. Moreover, for samples with higher Fe contents (H20Fe, H25Fe), activity decreases in parallel with the K content.

Concerning the influence of reducibility, the lack of correlation with total reducibility, as measured from the total hydrogen consumption in quantitative H_2_-TPR experiments (Figures 8 and 9), is clear. In the paper by Pan et al., a relationship has been suggested between low temperature reducibility, i.e., labile surface oxygen species, and activity. According to our quantitative analysis of H_2_-TPR profiles, Fe doping leads to a shift to lower temperatures of the α and β reduction events (Table S3, Figure S13a) up to H15Fe. Nevertheless, the oxygen-involved trend in these peaks steadily decreases (Figure S13b) up to Fe contents of 15%, and finally increases for the samples with the highest Fe contents.

Regarding reducibility under CO (Figure S11), changes in the Fe content affect mostly oxygen removal in the medium temperature range, whereas only small differences are observed at low temperatures. Therefore, taking into account these results and the fact that we are considering a comparison between room-temperature activity values, reducibility, considered as a total amount of oxygen available for exchange, does not seem to be the key parameter explaining activity modification in this family of hollandites. Only, the most labile character of surface oxygen species could be contributing to improvement in activity, but this is rather linked to the presence of Mn in a reduced state or of additional oxygen vacancies. In the paper by Pan et al., K content and Mn oxidation states were linked to each other, in such a way that separating the specific contribution of each factor could not be performed, but in this contribution, the two factors have been isolated.

Though the incorporation of Fe by itself cannot explain the evolution of activity along the whole series, as already commented, Fe^3+^ doping introduces different structural effects, in terms of Mn–O bonding in MnO_6_ octahedra, the presence of O–H bonds in the channels, and the modulation of the oxidation state of Mn. Thus, Fe incorporation reflects clearly in the total content of Mn^3+^ and oxygen vacancies, both of which reach a maximum value in H15Fe, the most active sample per surface area unit.

The high performance of **H15Fe** could then be due to a cooperative effect between its high amount of hydroxyl groups, in particular those inserted in the tunnels, and its lower average Mn oxidation state.

On the basis of a detailed characterization study, in which XAFS (XANES, EXAFS) and XRD were employed as structural analysis techniques, Ma et al.^58^ also pointed out the key role of Mn^3+^ content in the catalytic performance of metal-doped Mn hollandites in the ozone decomposition reaction. In the particular case of the Fe-doped catalyst investigated in this work, all Mn in the solid was present as Mn^4+^. This result, together with the K and Fe contents determined by ICP-OES, indicates that the hydrothermal one-step synthesis led in this case to a fully oxidized catalyst, with composition roughly K_0.10_Mn_0.89_Fe_0.11_O_2.00_, i.e., with no O vacancies present. In the Fe-doped catalysts investigated in our work, this is not the case for any of the samples, which always contain, as directly determined from ND experiments, a contribution of oxygen vacancies, which ranges from roughly 3% in the undoped sample up to 7% in the Fe-doped materials. These results point out the intrinsic complexity of these materials and reveal how the synthesis conditions may lead to subtle modifications in the oxygen sublattice, which, in any case, may be relevant in terms of catalytic performance.

The relative amounts of Mn^4+^ (d^3^) and Mn^3+^ (d^4^) may influence the system in two different ways: (1) modifying the Mn–O bond strength and, therefore, oxygen lability at surface positions and (2) dictating the occupancy of the e_g_ electronic states, for which e_g_ orbital occupancy by one electron (e_g_^1^) has been demonstrated as optimal for the catalytic activity in several processes.^88−92^ In particular, for CO oxidation,^88,91^ calculations have shown that CO is bonded with transition-metal atoms by the donation of lone pair electrons into the transition-metal e_g_ orbital, giving rise to a σ-bond molecular orbital, accompanied by back-donation of t_2g_ electrons to the π* antibonding orbital of CO, thus favoring CO oxidation. Therefore, the superior activity of the **H15Fe** sample correlates not only with its highest content of hydroxyl groups, particularly those inserted in the tunnels, but also with its highest content of Mn^3+^(e_g_^1^) species. In fact, a very good agreement is observed between the evolution with doping of catalytic activities at room temperature (Figure 6) and Mn^3+^ content (Figure S14)

## 4 Conclusions

A family of iron-doped hollandites, K*_x_*Mn_1–*y*_Fe*_y_*O_2−δ_ (0 ≤ *y* ≤ 0.15), with outstanding catalytic performance at room temperature in the CO oxidation have been synthesized. A detailed compositional and structural study of the cationic and anionic sublattices has been performed, which included a quantitative evaluation of the oxygen and hydroxyl contents, as well as of the average Mn oxidation state.

Atomically resolved EELS data directly proved the incorporation of Fe into the Mn positions, as substitutional specie, lowering the average Mn oxidation state and stabilizing hydroxyl groups within the hollandite tunnels as in isostructural β-FeOOH.

The reducibility of these oxides under H_2_ and CO correlates with Mn average oxidation state and becomes optimal for an actual Fe molar content of 12%, which is also the catalyst depicting the highest specific (per unit surface area) activity at room temperature.

The whole set of characterization results suggests a major influence of Mn^3+^ in the catalytic activity at a low temperature, though a secondary contribution of tightly bonded OH groups at the tunnels cannot be disregarded.

## Supporting Information Available

XRD, ND, TEM/STEM, TPO DRIFT, and CO-TPR/H_2_-TPR data (PDF)
