# Supplementary material for: Exceptional Low-Temperature CO Oxidation over Noble-Metal-Free Iron-Doped Hollandites: An In-Depth Analysis of the Influence of the Defect Structure on Catalytic Performance
Source: ACS Catal. 2021 Dec 1;11(24):15026–39. doi: 10.1021/acscatal.1c04954 (PMC8713355; doi:10.1021/acscatal.1c04954)
Supplement: Supplementary file 1 — cs1c04954_si_001.pdf [file cs1c04954_si_001.pdf]

Exceptional Low Temperature CO Oxidation over Noble Metal-Free Iron Doped Hollandites: an in-depth analysis of the influence of the defect structure on catalytic performance

Isabel Gómez-Recio[a], Huiyan Pan[b], Alberto Azor-Lafarga[a], María Luisa Ruiz-González[a], María Hernando[a], Marina Parras[a], María Teresa Fernández-Díaz[c], Juan J. Delgado[b], Xiaowei Chen[b], Daniel Goma Jiménez[b], David Portehault[d], Clément Sanchez[d], Mariona Cabero[e], Arturo Martínez-Arias[g], José M. González-Calbet[a,e,] and José J. Calvino[b,f,*]*

[a]Departamento de Química Inorgánica, Facultad de Químicas, Universidad Complutense, 28040-Madrid, Spain

[b]Departamento de Ciencia de los Materiales e Ingeniería Metalúrgica y Química Inorgánica, Facultad de Ciencias, Universidad de Cádiz, Campus Rio San Pedro, Puerto Real, Spain

[c]Institut Laue-Langevin, 38042, Grenoble cedex 9, France

[d]Sorbonne Université, CNRS, Collège de France, Laboratoire Chimie de la Matière Condensée de Paris, 4 Place de Jussieu, 75005 Paris, France

[e]ICTS ELECMI-Centro Nacional de Microcopia Electrónica, Universidad Complutense, 28040-Madrid, Spain

[f]ICTS ELECMI-DME Universidad de Cádiz, Campus Rio San Pedro, Puerto Real, Spain

[g]Instituto de Catálisis y Petroleoquímica, CSIC, Marie Curie 2, Cantoblanco, 28049-Madrid, Spain

Corresponding Author

* José J. Calvino

jose.calvino@uca.es

* José M. González-Calbet

jgcalbet@ucm.es

SI1. X-Ray Diffraction

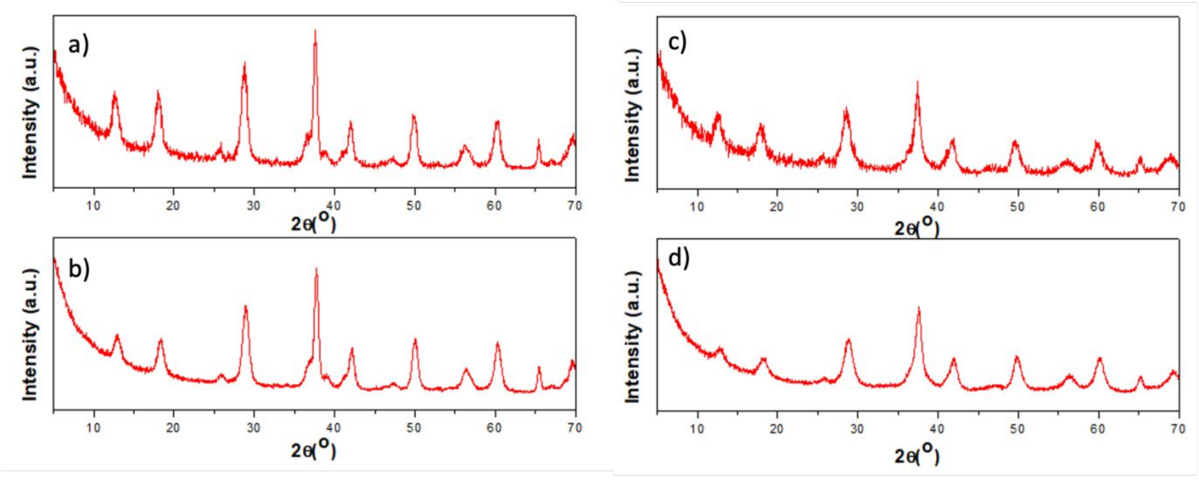

Figure S1. XRD patterns of H (a,b) and H15Fe (c,d) before and after the drying process (12 h at 120°C), respectively.

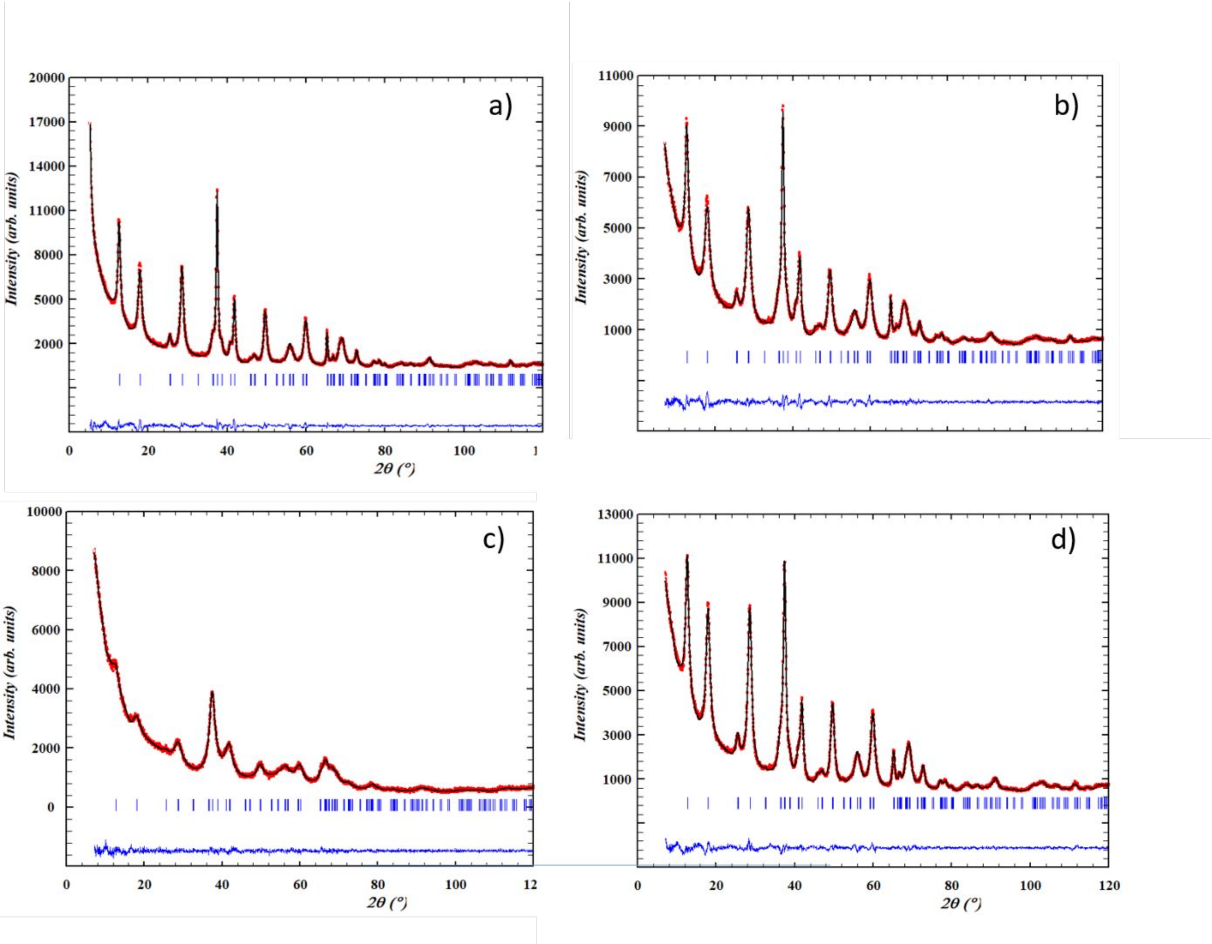

Figure S2. Le Bail profile fitting of X-Ray diffraction data of iron-doped hollandites ($K_nMn_{1-x}Fe_xO_{2-\delta}$), a) **H10Fe**, b) **H15Fe**, c) **H25Fe**, d) **H15Fe** obtained by a **two-steps synthesis**.

SI2. Neutron Diffraction results

Table S1. Structural parameters from the refinement of neutron powder diffraction data for **H15Fe**.

Mn/Fe (x, y, 0)	
X	0.3465(8)
Y	0.1692(8)
occ	a
K (0, 0, 0.5)	
occ	0.44
O1 (x, y, 0)	
X	0.1537(3)
Y	0.2027(3)
occ	1
O2 (x, y, 0)	
X	0.5425(3)
Y	0.1688(5)
occ	0.893(14)
H (x, y, 0.5)	
X	0.441(1)
Y	0.357(5)
occ	0.091(8)
I4/m, a = b = 9.8381(4) Å, c= 2.86373(9) Å	

$K_{0.11}Mn_{0.877(2)}Fe_{0.123(2)}O_{1.80(1)}(OH)_{0.09(1)}$

[a]Mn/Fe: 0.877(2)/0.123(2)

Fit parameters: $B_{overall}$=0.88(1), R_B=4.18, R_p=1.95, R_{wp}=2.46, χ^2=2.12

The OH composition is determined from the H occupation since to form OH group the H atoms is bonded to O1. The occupation of K was fixed to the composition obtained by EMPA.

Table S2. Selected Interatomic distances (Å) in iron-doped hollandite oxides from neutron powder diffraction data

	H15Fe
M-O1	1.924(8)
M-O1 x2	1.908(5)
M-O2	1.928(8)
M-O2x2	1.904(5)
O1-H	1.09(3)
M-M edge-sharing	2.861(10)<br>2.8637(1)
M-M corner-sharing	3.48(1)

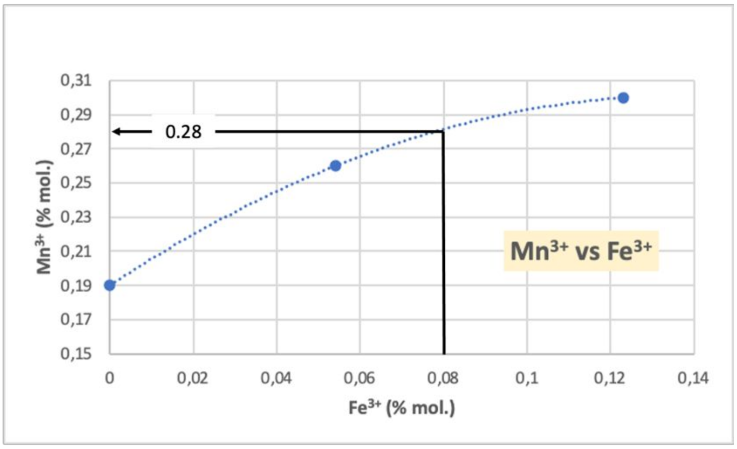

Figure S3. Interpolation of the Mn^{3+} content in the H10Fe catalyst, considering the Mn^{3+} vs Fe^{3+} data determined by ND for the H, H5Fe and H15Fe catalysts. According to ND, the Fe^{3+} content of H10Fe amounts to 0.08% mol.

SI3. TEM/STEM

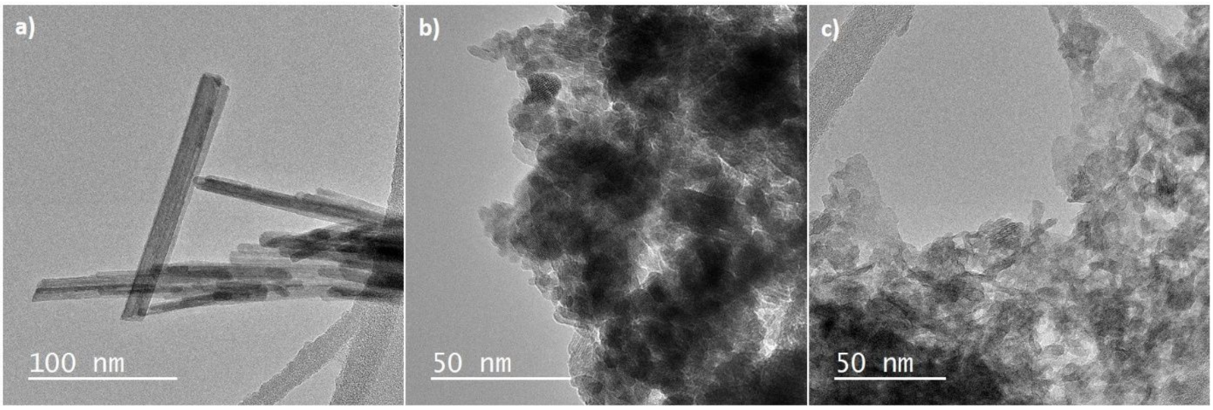

Figure S4. Characteristic low magnification TEM images for samples with different nominal Fe contents: a) **H**, b) **H15Fe** and c) **H25Fe**.

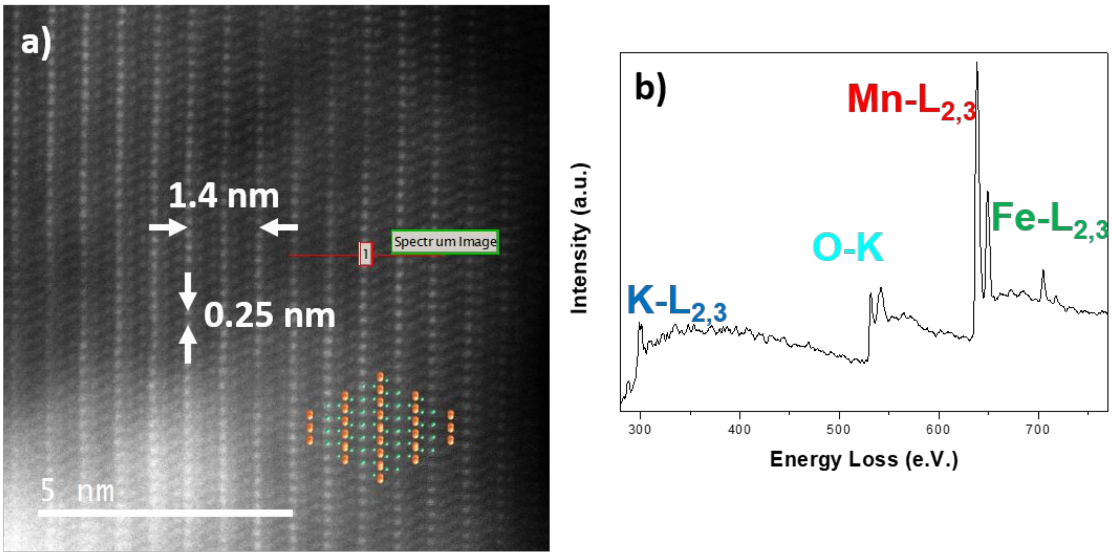

Figure S5. a) Characteristic STEM-HAADF image for sample **H15Fe** showing interatomic distances in agreement to the hollandite unit cell along [113] direction; b) EELS sum spectrum along the line marked in a.

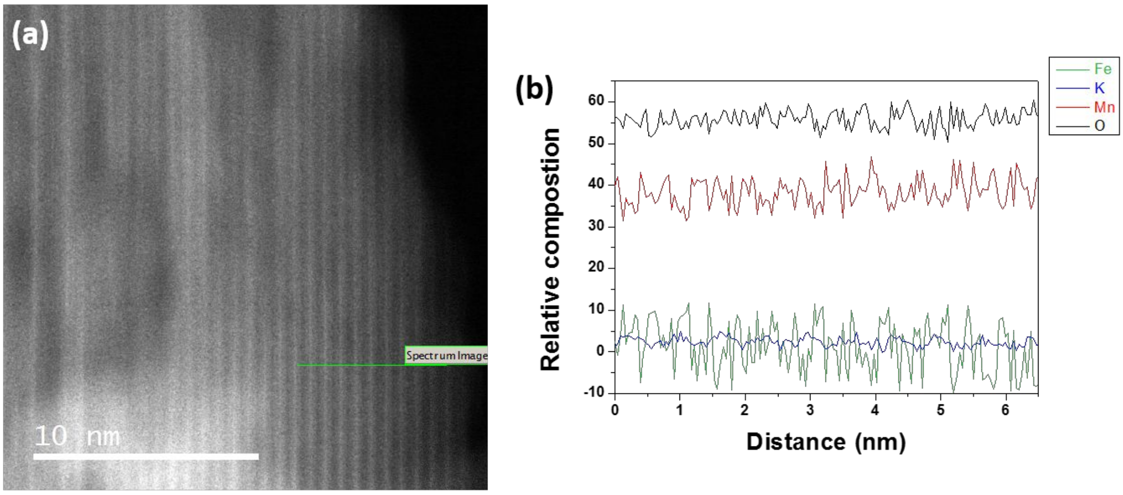

Figure S6. Results of EEL Spectrum Line experiments performed on H15Fe. (a) STEM-HAADF image of a H15Fe crystallite where a spectrum line (marked with yellow line) was acquired. The path extends from the surface to the bulk of the crystallite; (b) Relative composition in Fe, K, Mn and O along this line.

SI4. Study of catalyst pre-treatment conditions

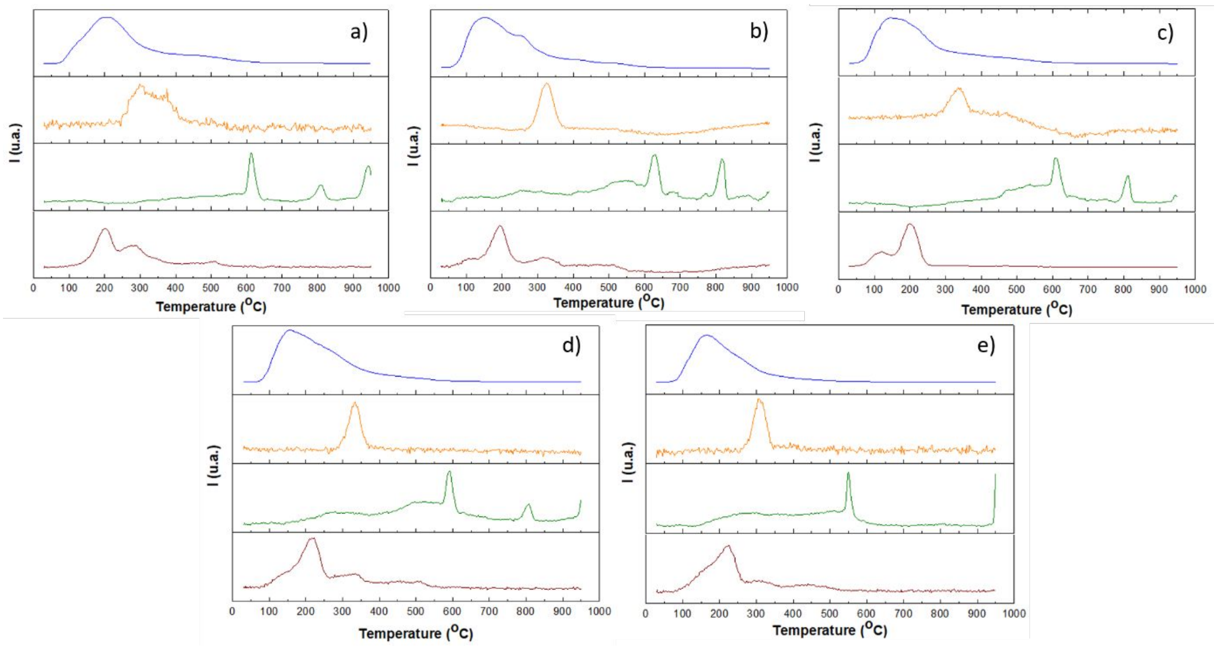

Figure S7. TPO-MS study of iron doped hollandites a) **H5Fe**, b) **H10Fe**, c) **H15Fe**, d) **H20Fe** and e) **H25Fe**. Color code: 18/4 blue, 30/4 orange, 32/4 green, 44/4 brown.

SI.5 DRIFT study

DRIFTS spectra under reaction conditions were recorded for the most active doped hollandite (sample H15Fe) and for the undoped material (sample H) intending to get hints on the CO oxidation catalytic mechanism. Differences are observed between both catalysts. Thus, the doped system (Figure S7) exhibits the formation of relatively broad bands at ca. 1595 and 1282 cm^{-1} along with a narrower one at ca. 1413 cm^{-1} already upon contact with the reactant mixture at the lower examined temperature (15 °C). The two former apparently disappear or transform to a wide band centred at ca. 1440 cm^{-1} at 50 °C while the band at 1413 cm^{-1} decreases although it is still clearly visible at that reaction temperature. All these bands apparently disappear at 100 °C or higher reaction temperature. In contrast, no band is formed in

the undoped sample examined under the same conditions and up to 300 °C reaction temperature (Figure S8). In turn, the CO conversion observed during the course of these experiments is displayed in Figure S9. Note, as observed during catalytic activity tests (Fig. 6a), the higher catalytic activity of H15Fe while differences in respective CO conversion levels achieved are related to the differences in the conditions employed in each case, most particularly the different type of reactor employed.

The bands detected appear in the range expected for carbonate species[1]. Spliting of the E vibration of the free carbonate ion (at ca. 1415 cm^{-1}) can be produced as a consequence of the symmetry lowering associated to carbonate coordination on the oxide surface while the magnitude of such splitting provides hints on the type of coordination of the carbonate on the surface [**Error! Bookmark not defined.**]. Thus, based on the evolution of the bands detected, the bands at ca. 1592 and 1282 cm^{-1} can be assigned to a bidentate carbonate while the band at 1413 cm^{-1} can correspond to a symmetric carbonate (in which the splitting of the degenerate E mode in two bands is not expected). The observed carbonates can be reaction intermediates for the $CO-O_2$ reaction, although we cannot discard them as simple spectators formed as a consequence of the adsorption of the CO_2 produced in the reaction. In any case, the results reveal the capability of the doped sample for forming carbonate species on its surface which could well favour a different reaction mechanism and explain its higher catalytic activity for CO oxidation.

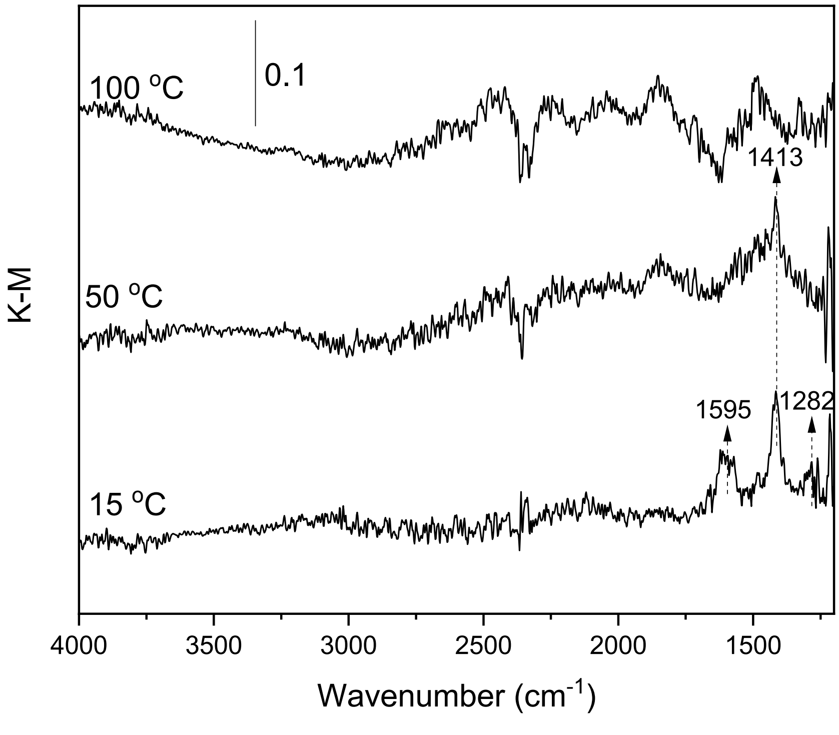

Figure S8. Difference DRIFTS spectra (using as subtraction reference the one recorded prior to introduction of the $CO-O_2$ reactant mixture) for sample H15Fe under 1% CO and 0.6% O_2 at the indicated temperatures.

[1] G. Busca, V. Lorenzelli. "Infrared spectroscopic identification of species arising from reactive adsorption of carbon oxides on metal oxide surfaces". Mater. Chem. 7 (1982) 89- 126

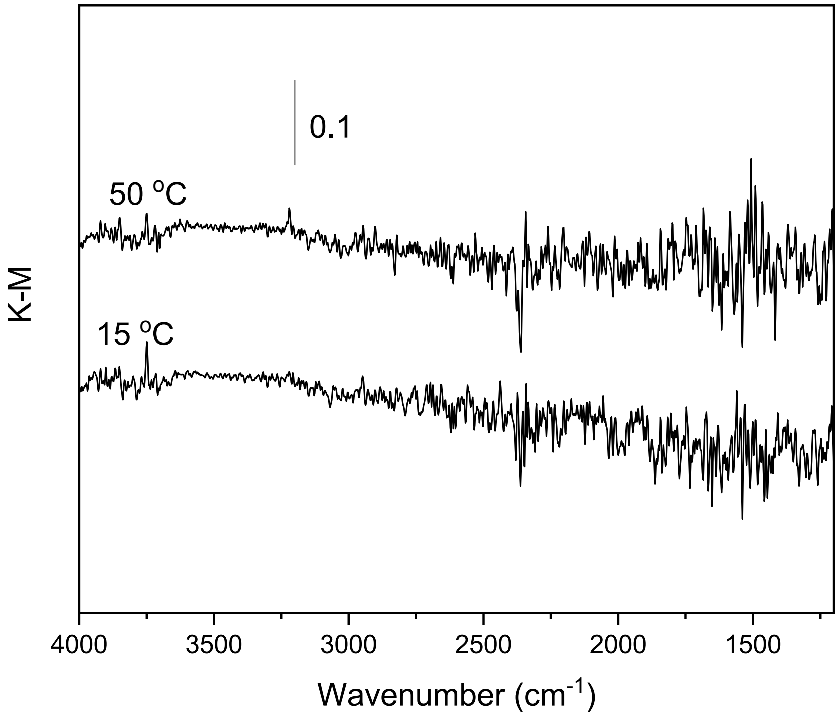

Figure S9. Same as Fig. 1 for sample H.

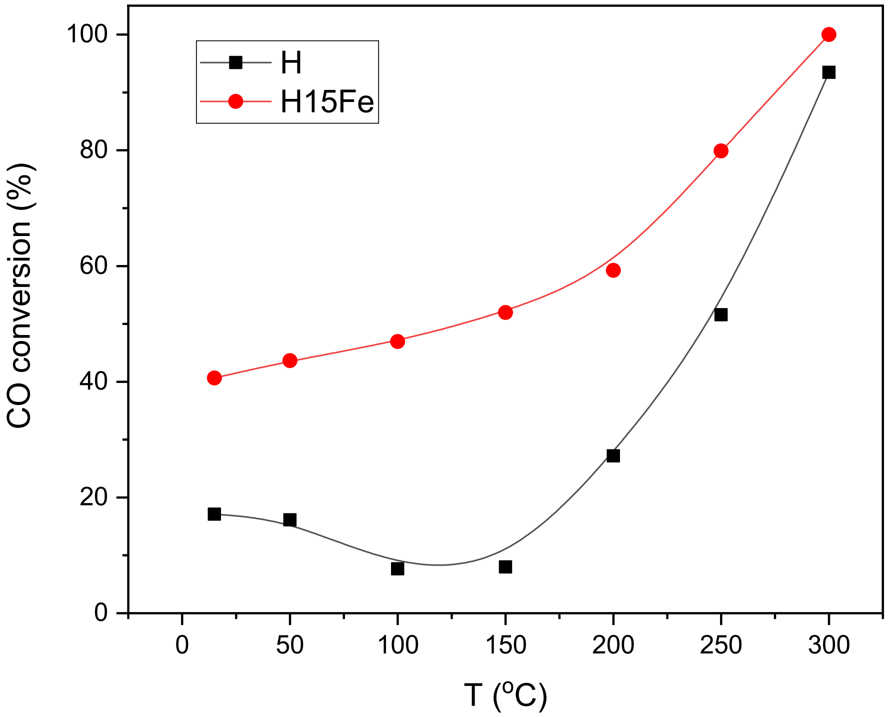

Figure S10. CO conversion during the DRIFTS experiments under CO-O_2 mixture.

SI6. Study of catalyst redox properties

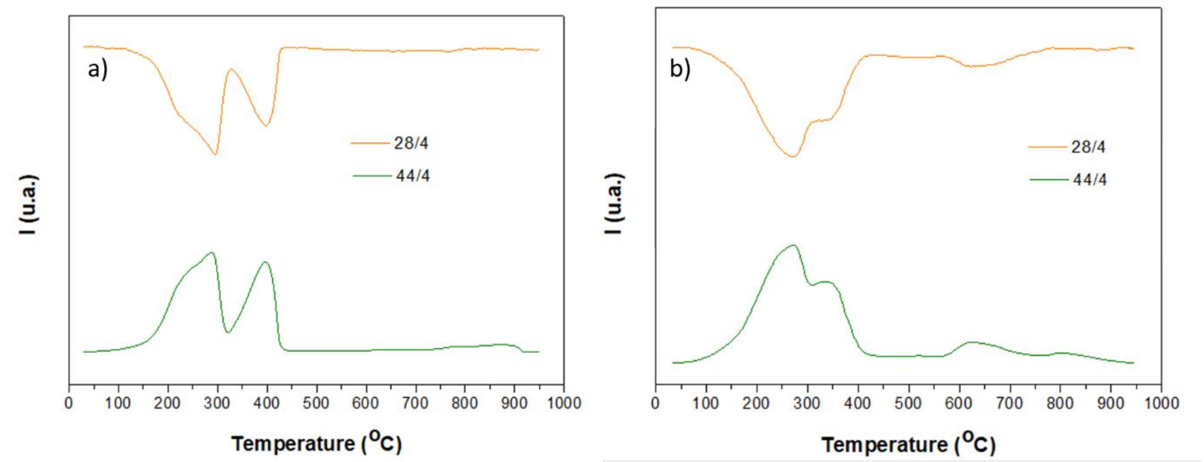

Figure S11. CO-TPR-MS study of a) undoped hollandite and b) iron-doped hollandite oxide **H15Fe**.

Table S3. H_2 consumption observed during H_2-TPR-TCD characterization of hollandite oxides. Temperature and integrated area (A%) of the deconvoluted peaks of TPR profile.

		1	2	3	4	5	6	7	H_2 consuption (mmol·g^{-1})
**H**	T (°C)	232(α)	285(β)	319(γ)	423(δ)	555(ε)			10.9406
	A (%)	17.54	52.52	24.93	1.52	4.48			
**H5Fe**	T (°C)	269(α)	298(β)	366(β´)	422(γ)	653	685		9.5847
	A (%)	13.19	8.08	45.14	27.24	4.8	1.55		
**H10Fe**	T (°C)	166(α)	242(β)	296(β´)	323(γ)	536	582		8.5849
	A (%)	4.53	25	29.87	37.79	1.22	1.58		
**H15Fe**	T (°C)	109(α)	163(β)	263(β´)	309(β’’)	360(γ)	539	593	8.0449
	A (%)	0.83	2.43	40.65	3.96	22.56	0.96	1.61	
**H20Fe**	T (°C)	219(α)	253(β)	290(β´)	359(β´´)	584(γ)			8.6349
	A (%)	27.8	19.44	13.83	35.83	3.1			
**H25Fe**	T (°C)	203(α)	249(β)	285(β´)	363(γ)	432	616	708	9.3340
	A (%)	20.72	26.75	5.01	27.61	15.74	1.27	2.91	

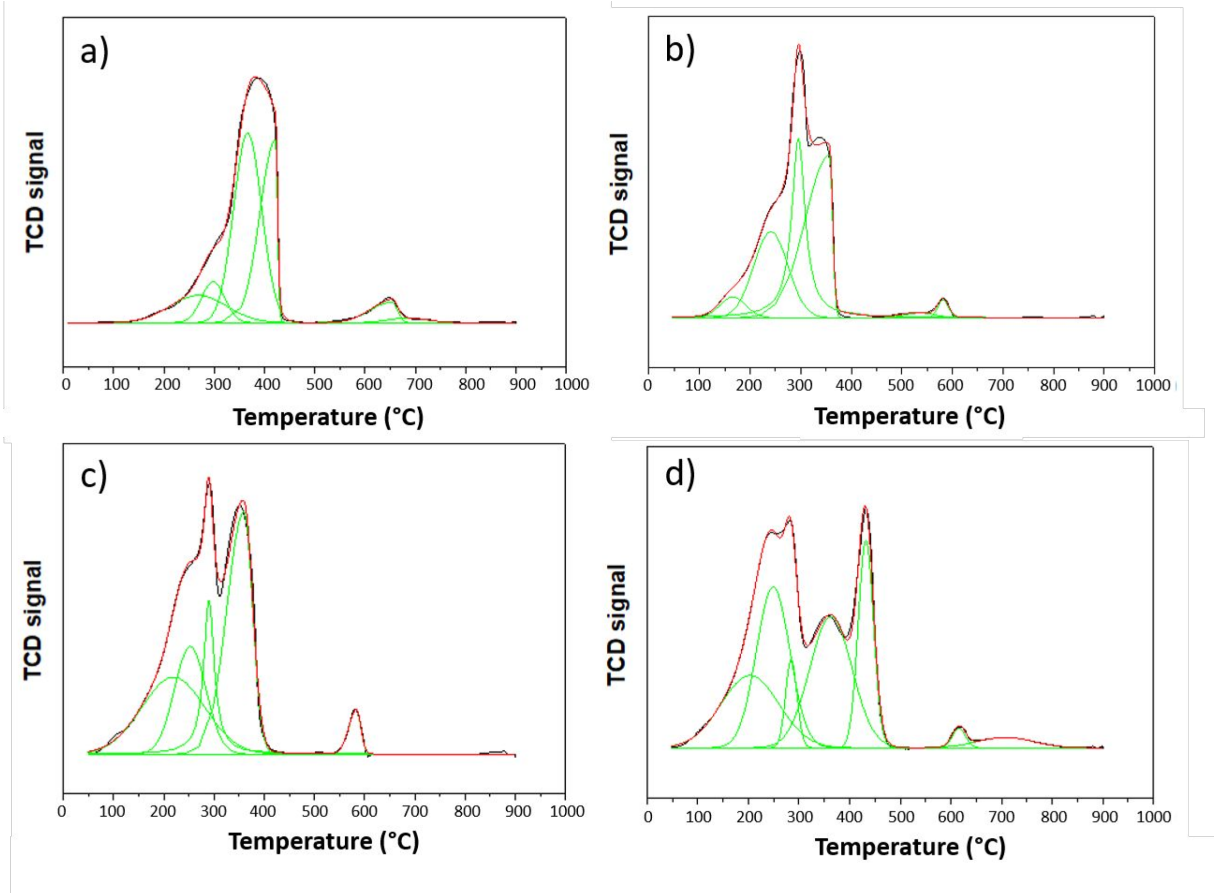

Figure S12. H_2-TPR-TCD study of iron-doped hollandites a) **H5Fe**, b) **H10Fe**, c) **H20Fe** and d) **H25Fe**.

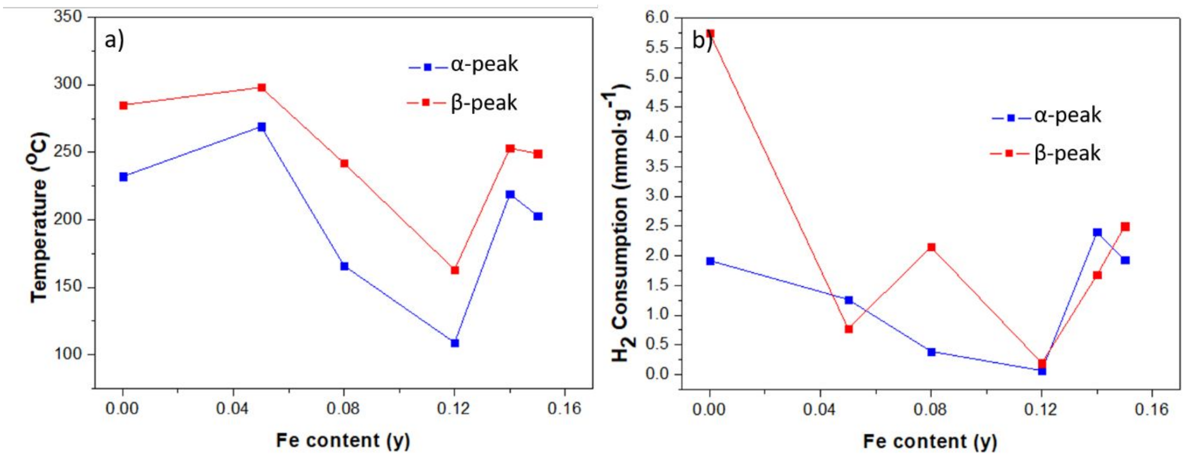

Figure S13. a) Hydrogen consumption and b) evolution of temperature values in α and β reduction events in H_2-TPR-TCD in iron doped hollandites ($K_xMn_{1-y}Fe_yO_{2-\delta}$).

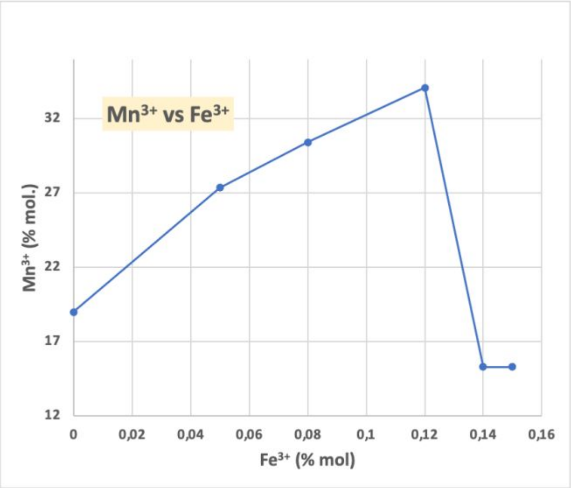

Figure S14. Evolution of Mn^{3+} content with Fe doping
